# Recalling the Biological Significance of Immune Checkpoints on NK Cells: A Chance to Overcome LAG3, PD1, and CTLA4 Inhibitory Pathways by Adoptive NK Cell Transfer?

**DOI:** 10.3389/fimmu.2019.03010

**Published:** 2020-01-09

**Authors:** Pilar M. Lanuza, Cecilia Pesini, Maykel A. Arias, Carlota Calvo, Ariel Ramirez-Labrada, Julian Pardo

**Affiliations:** ^1^Immunotherapy, Inflammation and Cancer, Aragón Health Research Institute (IIS Aragón), Biomedical Research Centre of Aragón (CIBA), Zaragoza, Spain; ^2^Instituto de Carboquímica ICB-CSIC, Zaragoza, Spain; ^3^Medical Oncopediatry Department, Aragón Health Research Institute (IIS Aragón), Hospital Universitario Miguel Servet, Zaragoza, Spain; ^4^Unidad de Nanotoxicología e Inmunotoxicología (UNATI), Centro de Investigación Biomédica de Aragón (CIBA), Aragón Health Research Institute (IIS Aragón), Zaragoza, Spain; ^5^Aragón i + D Foundation (ARAID), Government of Aragon, Zaragoza, Spain; ^6^Department of Microbiology, Preventive Medicine and Public Health, University of Zaragoza, Zaragoza, Spain; ^7^Nanoscience Institute of Aragon (INA), University of Zaragoza, Zaragoza, Spain

**Keywords:** NK cell, adoptive cell therapy, T cell, immune check point, cancer

## Abstract

Immune checkpoint receptors (IC) positively or negatively regulate the activation of the host immune response, preventing unwanted reactions against self-healthy tissues. In recent years the term IC has been mainly used for the inhibitory ICs, which are critical to control Natural Killer (NK) and Cytotoxic CD8^+^ T cells due to its high cytotoxic potential. Due to the different nature of the signals that regulate T and NK cell activation, specific ICs have been described that mainly regulate either NK cell or T cell activity. Thus, strategies to modulate NK cell activity are raising as promising tools to treat tumors that do not respond to T cell-based immunotherapies. NK cell activation is mainly regulated by ICs and receptors from the KIR, NKG2 and NCRs families and the contribution of T cell-related ICs is less clear. Recently, NK cells have emerged as contributors to the effect of inhibitors of T cell-related ICs like CTLA4, LAG3 or the PD1/PD-L1 axes in cancer patients, suggesting that these ICs also regulate the activity of NK cells under pathological conditions. Strikingly, in contrast to NK cells from cancer patients, the level of expression of these ICs is low on most subsets of freshly isolated and *in vitro* activated NK cells from healthy patients, suggesting that they do not control NK cell tolerance and thus, do not act as conventional ICs under non-pathological conditions. The low level of expression of T cell-related ICs in “healthy” NK cells suggest that they should not be restricted to the detrimental effects of these inhibitory mechanisms in the cancer microenvironment. After a brief introduction of the regulatory mechanisms that control NK cell anti-tumoral activity and the conventional ICs controlling NK cell tolerance, we will critically discuss the potential role of T cell-related ICs in the control of NK cell activity under both physiological and pathological (cancer) conditions. This discussion will allow to comprehensively describe the chances and potential limitations of using allogeneic NK cells isolated from a healthy environment to overcome immune subversion by T cell-related ICs and to improve the efficacy of IC inhibitors (ICIs) in a safer way.

## Introduction

**Figure 1:**
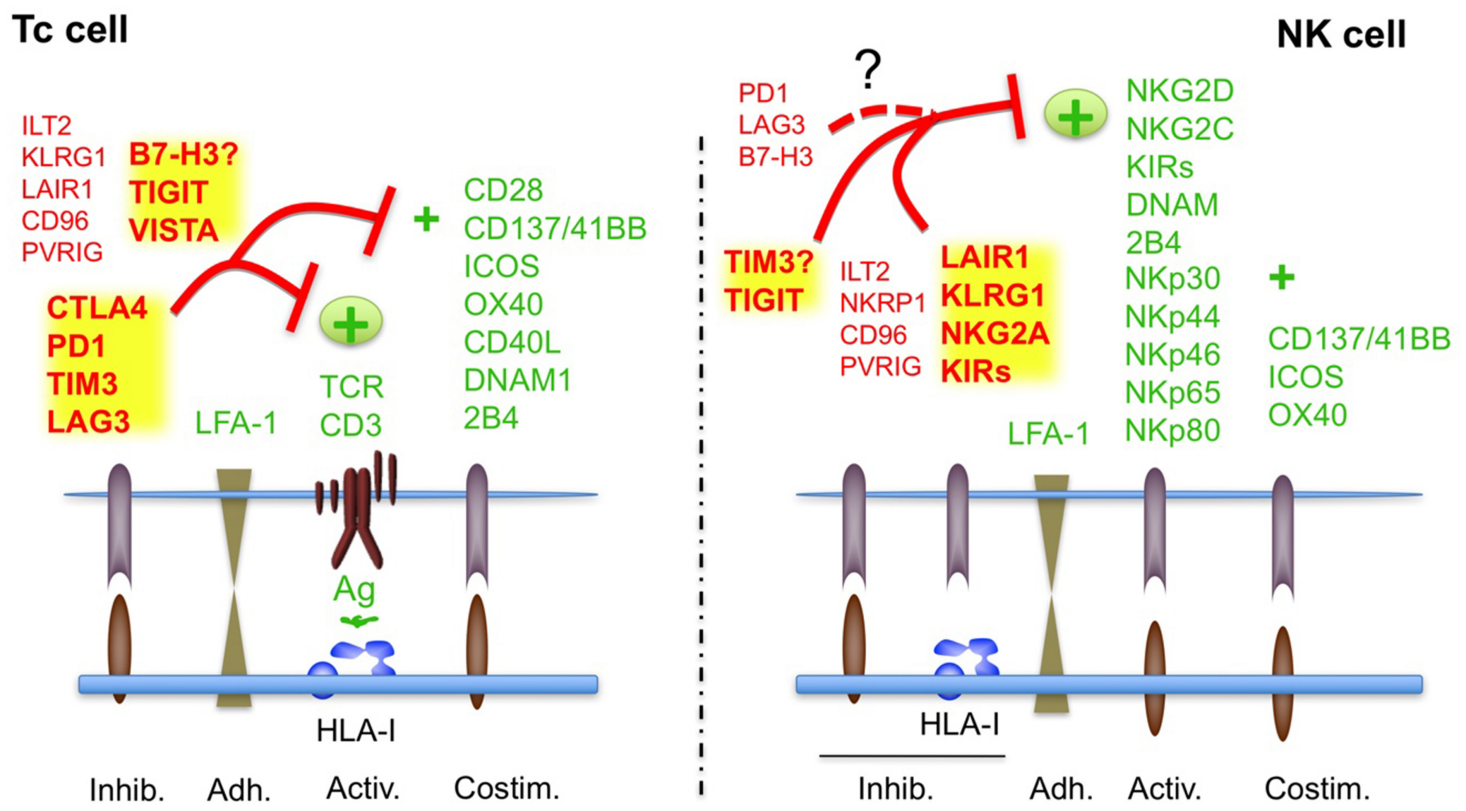
Regulation of Tc and NK cell activity by ICs in physiological conditions. The canonical ICs, involved in tolerance are indicated in bold. See also Tables 1, 2. Receptors with no clear functions are indicated with?. It is not clear if B7H3 plays an inhibitory or activating role in T cell tolerance; TIM has been shown to inhibit or activate NK cells.

Natural Killer (NK) cells are a class of innate lymphocytes that have evolved to eliminate rapidly infected and tumor cells. In humans there are two main subsets, phenotypically and functionally different, classified phenotypically according to the level of membrane expression of CD56 and CD16 and functionally according to their cytotoxic potential (1, 2). The CD56^dim^CD16^pos^ NK cell subset is eminently cytotoxic and expresses high levels of perforin and granzyme B. They share similar immunosurveillance function and killing mechanisms with the other main cell population responsible for killing infected or tumor cells, cytotoxic CD8^+^ T (Tc) cells. However, mature NK cells can exert their cytotoxic function without previous activation and independently of the presence of non-self antigens presented by MHC molecules. Despite the ability of NK cells to eliminate target cells without previous sensitization, it is now well-known that previous activation enhances NK cell activity by regulating the expression of both cytotoxic mediators and several receptors as explained below (Figure 1) (3, 4). Moreover, a previous exposition to specific antigens (haptens), viral infection (CMV) or cytokines (IL12, IL15, and IL18) generates a type of NK cells known as adaptive NK cells that possess immunological memory features (5). However, the mechanisms that regulates the generation of adaptive NK cells are not clear yet and, specifically, it is not known if the HLA-self peptide complexes play any role in this process (6). However, previous results suggest that, at least in mouse models, this is not the case since mature NK cells are able to proliferate when transferred into MHC-I deficient recipients (7).

Since the activity of mature educated NK cells does not depend on neoantigens presented by MHC, they might be the perfect candidates to develop therapeutic strategies to eliminate tumors that are not visible for T cells either because they have downregulated MHC/HLA-I or because they do not present “good” HLA-I associated antigens. However, as it will be discussed below, since the key steps involved in the regulation of T and NK cells are different, it is very difficult to extrapolate the wide knowledge acquired on T cell regulation to NK cells, including the function of some inhibitory immune checkpoints (ICs) like CTLA-4 and PD1. Indeed, the development of specific immunotherapy protocols based on NK cells to treat cancer has been dampened by the complexity of the mechanisms that regulate NK cell function and elimination of target cells. Luckily, times are changing and, at present, in the era of cancer heterogeneity and immunotherapy, NK cells are emerging as the golden boys to eliminate non-antigenic tumor cell clones. A perfect duet in the symphony of destruction, Tc and NK cells destroying immune “visible” and “invisible” cancer cells to overcome immunogenic tumor heterogeneity.

However, to fully exploit the potency of this consortium in a safe way, it is required to understand in more detail the role of ICs in the regulation of Tc and/or NK cells during physiological immune responses as well as in the cancer microenvironment. As indicated in the summary, conceptually IC could be defined as any molecule involved in the regulation of the immune response, either in a positive or negative way. However, for reasons of clarity, in this review the term IC will be used to refer to the inhibitory ICs, unless the contrary is indicated.

## The Canonical Immune Checkpoints of NK Cells (NK cell-ICs)

The mechanisms involved in the regulation of NK cell self-tolerance and immunosurveillance against damaged cells has been by far much less understood that those regulating Tc cell activity (8). However, the knowledge accumulated during the last two decades has allowed to reveal a plethora of receptors involved in the regulation of NK cell activation and elimination of tumor cells. These receptors are known as ICs of NK cells (NK cell-ICs), since they are involved in the regulation of NK cell tolerance against healthy tissues, while ensuring an efficient response against damaged cells (Table 1). Thus, according to the physiological function of ICs, they could be considered as canonical ICs. Among others, NKG2A, KIRs and 2B4 are considered as canonical NK cell-ICs and have been proposed to regulate NK cell self-tolerance, also known as NK cell education (9, 10). It should be noted here that 2B4 can act either as an inhibitory or activating NK cell receptor (11). For example, inhibitory functions have been reported in mouse NK cells (12), human NK cell progenitors (9), and X-linked lymphoproliferative syndrome patients (13). In mature human NK cells, 2B4 mainly acts as an activating receptor (Figure 1). NK cell education is also regulated by the activating NK cell receptors SLAM, NKG2C, NKG2D, and NKp46 (9, 10).

**Table 1:** Canonical human NK cell checkpoints.

**Receptor**	**CD**	**Effect**	**Other cell types**	**Ligand**	**Tolerance^a^**	**Induced/Constitutive**
KIR-L	CD158	Inh	T, Tγδ, Tm	HLA- I	Yes	C
CD94/NKG2A	CD159a	Inh	T CD8^+^, Tγδ	HLA-E	Yes	C
ILT2	CD85j	Inh	T, B, Tm	HLA-A, B, C, HLA- G UL18	?	C
KLRG-1	-	Inh	ILC2, T	Cadherins	Yes^b^	C
LAIR-1	CD305	Inh	T, B, myeloid	Collagen	Yes^b^	C
NKRP1A	CD161	Inh	T, NKT, ILC	LLT1	?	C
Fc?RIII	CD16	Act	T, myeloid	IgG-Fc	Yes	C
KIR-S	CD158	Act	T, Tγδ, Tm	HLA-I	Yes	C
NKp30	CD337	Act	ILC2, T, Tγδ c	B7H6, BAG6/BAT3	Yes	C
NKp44	CD336	Act	ILC3, myeloid	MLL5 Nidogen-1 HLA-DP	?	I
NKp46	CD335	Act	-	Properdin, HA, HN	Yes	C
NKp65	-	Act	-	KACL	?	C
NKp80	-	Act	T CD8^+^, Tγδ	AICL	?	C
NKG2D	CD314	Act	T CD8^+^, Tγδ	MICA/B, ULBPs	Yes	C
CD94/NKG2C CD94/NKG2E	CD159c CD159e	Act	T CD8^+^, Tγδ	HLA-E	Yes	C
2B4	CD244	Act/Inh^c^	T, Tγδ, granulocyte	CD48	Yes	C
DNAM-1	CD226	Act	T, B, granulocyte	CD112 (Nectin-2), CD155 (PVR)	No	C
41BB	CD137	Act	T, myeloid, endothelial, tumor	CD137L	No	I
ICOS	CD278	Act	T	ICOS-L B7RP-1	No	I
OX40	CD134	Act	T, NKT granulocyte	OX40-L (CD252)	No	I

*HA, Hemagglutinin; HN, Hemaglutinin-Neuraminidase; LFA, Lymphocyte function-associated antigen 1; MICA/B, Major histocompatibility complex (MHC) class I chain-related protein A/B; MLL5, Mixed lineage leukemia 5; Tm, Memory T cell; PVR, Poliovirus receptor; ULBP, UL16-binding protein; C, constitutive; I, induced*.

a *Regulation of human NK cell education and/or tolerance*.

b *Role in NK cell tolerance is controversial*.

c *2B4 is an activating receptor in human mature NK cells but an inhibitory receptor in mouse NK cells, human NK cell precursors during NK cell differentiation and in X-linked lymphoproliferative disease (XLP) patients*.

*? No reported function on NK cell tolerance*.

In contrast, the role of other ICs, which are known to regulate T cell-tolerance and autoimmunity like PD1, CTLA4, or LAG3 (T cell-ICs, Figure 1), in the control of NK cell tolerance is less clear, and, thus, they should not be considered as canonical ICs of NK cells, at least under non-pathological healthy conditions. Indeed, the expression of most of them is very low or absent in most NK cells from healthy patients (14, 15). A notable exception is the expression of PD1 that has been identified in a subset of mature NK cells which share features of adaptive memory-like NK cells (16). This subset is produced by individuals (around 25% of healthy people) previously exposed to CMV infection (17). The role of these non-canonical NK cell-ICs in the function of healthy NK cells will be discussed in more detail below.

### Regulation of NK Cell Activity by NK Cell-ICs

The net balance between inhibitory and activating signals transduced by the respective receptors will dictate if a NK cell kills or respect a target cell (Figure 1) (3, 4).

The inhibitory receptors/ICs mostly interact with HLA-I molecules and on this way they can sense transformed cells that have downregulated HLA-I, which makes them invisible for T cells (Figure 1). The main NK cell-ICs are members of the KIR (Killer-cell immunoglobulin-like receptor) family, NKG2A and ILT2 that recognizes HLA-A/B/C, HLA-E/G, and HLA-G haplotypes, respectively (18–20) (Table 1). As indicated in Table 1 and Figure 1, other inhibitory receptors modulate NK cell activity, albeit they have not been considered as ICs, since its role in NK cell tolerance and education is not clear.

Concerning activating receptors (Table 1), the family of Natural Cytotoxicity Receptors (NCRs), mainly NKp30, NKp44, and NKp46 (21) recognize different ligands and NKG2D recognizes stress ligands of the MIC family (22). Some members of the KIR family act as activating receptors when binding to specific HLA-C and HLA-A proteins and to HLA-G (23). NKG2C is an activating receptor for HLA-E (19, 20). NK cells also express the activating low affinity IgG receptor (FcγRIII), CD16 that mediates Antibody Dependent Cellular Cytotoxicity (ADCC) and the activating receptor DNAM-1 that binds to different ligands (Table 1). Finally, the C-type lectin receptors NKp65 and NKp80 also participates in NK cell activation after interacting with their respective ligands of the CLEC family, KACL, and AICL (Table 1) (24). NK cells also require adhesion and co-stimulatory proteins, like LFA-1 or 2B4 to eliminate cancer cells (25).

The only activating receptor that has not been found in non-activated NK cells is NKp44 that is rapidly upregulated after *in vitro* cytokine-mediated activation (26). Although NKp44 has been found to be constitutively expressed in a tissue-specific fashion on type 3 innate lymphoid cells and a subset of DCs (27), the role of this receptor in tumor immunosurveillance is not clear since it has not been detected yet in circulating or tumor infiltrated NK cells *in vivo*. Thus, in contrast to most NK cell-ICs and activating receptors, NKp44 has not been tested yet as a candidate for immunotherapy (28). It should be noted that an isoform of NKp44 can act as inhibitory receptor after interacting with the Proliferating Cell Nuclear Antigen (PCNA) (29). Indeed, it has been recently shown that an antibody against PCNA enhances the anti-tumoral effect of NK cells in a mouse model (30), suggesting that NKp44 acts as an IC in cancer. However, it is not known yet whether this isoform is a canonical NK cell-IC, regulating NK cell function in healthy individuals.

It has been recently described that some of these receptors like NKG2A, NKG2D, or NKp30 are also expressed by some T-cell subsets (31–33), although their expression in T cells from healthy individuals seems to be less frequent and they do not regulate T cell peripheral tolerance. Thus, its action might be restricted to pathological conditions like infection or cancer. Indeed, NKG2A has been recently shown to regulate both NK- and Tc cell-mediated anti-tumoral immunity (34, 35).

Reciprocally, as indicated above, it could be speculated that the regulatory activity of some ICs involved in the regulation of T cells like PD1, LAG3, CTLA4, or B7H3 is less pronounced in NK cells than in T cells (Figure 1). Supporting this hypothesis several studies have found that, in healthy individuals, CTLA4 is only expressed at very low levels in the cytosol of NK cells and PD1 or LAG3 could be restricted to specific memory-like subsets of individuals previously exposed to CMV infection (16, 36).

Other inhibitory receptors like ILT2, TIGIT, TIM-3, PVRIG, and TACTILE (CD96) might share regulatory functions in both NK and T cells, behaving as ICs in both cell types, although in this case their function in regulating NK cell tolerance is less clear (37, 38).

The contribution of these non-canonical emerging ICs to the regulation of healthy NK cell anti-tumoral activity will be discussed in more detail in section Allogeneic NK Cells Beyond KIR-Ligand Mismatch-Driven Alloreactivity: The Emerging Inhibitory NK-ICs.

### Modulation of Canonical ICs for Cancer Immunotherapy

In light of the biological relevance of ICs, regulation of self-tolerance vs. elimination of infected or transformed cells, it could be possible to differentiate between Tc cell- and NK cell-ICs, at least under physiological conditions (Figure 1). Thus, pharmacological or biological manipulation of NK cell-ICs has been mainly focused on low immunogenic tumor types that do not respond to T cell-based immunotherapy and for which the immunomodulatory role of some Tc cell-ICs like PD1 might be less pronounced (28). Especially on hematological cancers, where NK cells occupy the same niches as tumor cells (39).

As indicated above, the increased knowledge about the receptors regulating NK cell activity and cancer immunosurveillance is allowing the development of therapeutic approaches to increase NK cell activity against cancer cells. These protocols involve mono- and bi-specific antibodies against inhibitory or activating NK cell-ICs or against membrane tumor antigens (14, 28) as well as adoptive cell therapy using autologous or allogeneic NK cells (28).

Monospecific antibodies against membrane tumor antigens promote ADCC by engaging the activating receptor CD16 on NK cells. Among all the activating receptors it has been shown CD16 engagement is one of the most potent signals to activate NK cells and eliminate tumor target cells (40).

Antibodies against inhibitory KIRs were developed and its efficacy validated in mouse xenograft lymphoma models (41, 42). Subsequently they have entered clinical phase in acute myeloid leukemia, lymphoma, and some solid tumors. Pending of finishing the clinical trials, it seems that the anti-tumoral activity of these antibodies as monotherapy seems to be very low. However, the combination with chemotherapy or anti-PD1 antibodies might be more promising (43, 44).

Other antibodies that have been developed include anti-NKG2A (35) or bi- and tri-specific antibodies engaging NKp46 and/or CD16 and a tumor antigen (45, 46).

Only anti-NKG2A is currently being tested in clinical trials as monotherapy or in combination with anti-EGFR or anti-PD1 antibodies. Although the trial is not finished yet the results combining anti-NKG2A and anti-EGFR seem to be promising (35).

Albeit most clinical trials testing the antibodies against NK cell-ICs are still ongoing, the preliminary data indicate that they are not as efficient as the inhibitors of T cell-ICs, PD1, and CTLA4, neither against hematological nor against solid cancer (39). Regarding hematological cancer, this is likely due to that engaging a single IC is not enough to overcome the unregulated balance of signals received by NK cells on cancer patients. This is not easy to overcome since simultaneous engagement of several NK-ICs might lead to break down self-tolerance and induce toxicity. Among the potential consequences of targeting NK cell-ICs, it should be taken into account the implications of the findings by Carlsten et al. showing that inhibition of KIR2D by IPH2101 antibody impacts NK cell education (47). The authors showed a rapid decline in the function of NK cells from myeloma patients treated with IPH2101, which was related to the reduction of KIR2D expression on NK cell surface through monocyte trogocytosis. This treatment would affect the generation of educated NK cells with anti-tumoral activity due to the absence of interaction between KIR2D and its HLA-ligand (48). Thus, it might have a negative impact on other treatments designed to enhance NK cell activity by regulating ICs.

In the context of solid tumors, in addition to those problems, the lack of infiltration of NK cells and/or the immunosuppressive components of the tumor microenvironment might also contribute to the low efficacy of antibodies against NK cell-ICs (49).

## Adoptive Allogenic NK Cell Therapy: KIR-KIR Ligand Mismatch and NK Cell Alloreactivity

An ingenious way to overcome the limitations of antibodies against NK cell-ICs is the use of allogeneic NK cells: NK cells isolated from healthy haploidentical donors, which are transferred to patients un-manipulated or after *in vitro* activation and expansion. The question that allogeneic NK cells could efficiently kill tumor cells was addressed by Velardi et al., soon after discovery of the HLA-I inhibitory ligands of the KIR family. This finding indicated that NK cells are able to sense and response against missing-self or missing-HLA-I (50), due the loss of inhibitory signals transduced by inhibitory KIRs (51). Thus, it was found that NK cells generated in the host after haploidentical bone marrow transplantation presented alloreactivity against recipient leukemic cells (52), a process known as KIR-ligand mismatch. The clinical benefit of this alloreactivity was subsequently confirmed in acute leukemia patients undergoing allogenic bone marrow transplantation. Specifically, those patients that received a transplant from an haploidential donor and, thus, presented NK cell alloreactivity, prevented leukemia relapse (53). This finding was further confirmed by Miller's group (54).

Subsequently, different protocols to activate and expand allogenic NK cells from healthy haploidentical donors were developed and infusion of purified NK cells was tested in leukemia, lymphoma, and myeloma patients as well in solid tumors with different results (55, 56). In general, these clinical trials confirm a benefit of KIR-ligand mismatch in acute myeloid leukemia patients, yet there are number of factors affecting the effectivity of this protocol which have not been completely clarified. Among them, it is noteworthy to mention the selection of donors expressing specific KIR-ligand mismatched combination and the functional expression of KIRs on the membrane of NK cells. In addition, it is becoming evident the importance of selecting an adequate conditioning protocol, not only to prepare the recipient of the transplant, but also during the preparations of NK cells to be infused in the patients. For example, development of protocols that remove specific cell populations that inhibit NK cell activity like T regulatory cells (55, 57–59).

## Allogeneic NK Cells Beyond KIR-Ligand Mismatch-Driven Alloreactivity: The Emerging Inhibitory NK-ICs

### Biological Significance of T Cell-Related ICs: the Emerging NK Cell-ICs

Despite the unsolved questions in the clinical application of adoptive NK cell therapy, allogeneic NK cells might present several advantages over therapeutic manipulation of host NK cells. These advantages go beyond alloreactivity due to missing HLA-I inhibitory ligands. Specially, it should be stressed that allogeneic NK cells are selected from a healthy host and are not under the negative influx of cancer manipulation of host immunity. Thus, it is tempting to speculate that its action might not be restricted by ICs employed by cancer cells to overcome host Tc and NK cell anti-tumoral activity (28).

ICs were discovered in T cells by its ability to regulate T cell tolerance against self-antigens. Thus, both ICs and co-stimulatory signals have been shown to be involved in the regulation of T cell tolerance and prevention of autoimmunity (Figure 1) (15, 60). Subsequently, it was hypothesized that cancer cells might take advantage of the regulatory function of ICs to overcome T cell-immunosurveillance and, thus, pharmacological manipulation of ICs could be used as an effective cancer immunotherapy. The best example of this evolution is CTLA-4 and PD1 ICs. Both regulate T cell tolerance, preventing self-damage (61–64), and its inhibition by specific antibodies can reverse T cell inactivation (65) and is effective against specific cancer types (66, 67).

In contrast to NK cells, the regulation of T cell activity is restricted by their ability to differentiate self from non-self antigens and, thus, control of tolerance by inhibitory and co-stimulatory signals should be different in T and NK cells (Figure 1) (15, 60–64). In this context, meanwhile NK cells mainly counterbalance the activating signals by the presence of KIRs and other inhibitory ligands recognizing HLA-I (9, 10), T cells require other inhibitory signals to prevent TCR signaling by HLA-I-associated self-antigens and/or co-stimulation. Here the presence of specific T cell-ICs would contribute to self-tolerance, avoiding autoimmune reactions.

### Expression and Function of Emerging NK Cell-ICs in Cancer and Healthy Individuals

As indicated above this picture might be different under pathological conditions like cancer, and in those circumstances, some ICs traditionally associated to T cell function might promote NK cell immune-evasion by cancer cells (Table 2). Thus, although NK cells might be important players that contribute to the anti-tumor activity of IC inhibitors (ICIs) like antibodies against CTLA-4, LAG3, and the PD1/PD-L1 axes in cancer patients (68), this should not overestimate the function of some T cell-ICs in healthy NK cells. Indeed, most of Tc-ICs are not expressed by NK cells from healthy individuals even after activation and the level of expression of those that have been found is much lower than in T cells. These findings, key to develop efficient protocols and to exploit all the potential of adoptive allogeneic NK cell therapy, have passed unnoticed in the NK cell field. Indeed, most studies analyzing the expression of Tc-ICs in healthy NK cells are restricted to the use of healthy NK cell controls when analyzing expression of ICs in NK cells from cancer patients.

**Table 2:** Emerging human NK cell checkpoints.

**Receptor**	**CD**	**Effect**	**Other cell types**	**Ligand**	**Tolerance^a^**	**Induced/Constitutive**
B7H3	CD276	Inh	T, B, Treg, myeloid, non-immune cells, tumor	-	No	I
TIGIT	CD226	Inh	T, NKT	PVR (CD155) Nectin-2/-3 (CD112, 113)	Yes	C
TIM3	CD366	Act/ Inh	T, NKT, myeloid	Gal-9, HMGB1, PS, CEACAM1	Yes	C
LAG3	CD223	Inh	T, NKT, Treg, B	HLA-II, L-sectin, FGL1	No	I
PD1	CD279	Inh	ILC-2, T, B, NKT, myeloid	PD-L1, PD-L2	No	I
CTLA4	CD152	Inh	T, Treg	B7-1, B7-2	No	Unknown
PVRIG	CD112R	Inh	T	PVRL2 Nectin-2 (CD112)	?	C
TACTILE	CD96	Act/Inh?	T, NKT	PVR (CD155) Nectin-1 (CD111)	?^b^	C

*CEACAM1, Carcinoembryonic antigen-related cell adhesion molecule 1; FGL1, Fibrinogen-like protein 1; ILC, Innate Lymphoid cell; Gal-9, Galectin 9; HMGB1, High mobility group box 1; PS, Phosphatidylserine; Treg, regulatory T cell; C: constitutive, I: induced*.

a *Regulation of human NK cell education and/or tolerance*.

b *The role of CD96 in NK cell education/tolerance is not clear. It has been found that NK cells from mice deficient for CD96 are hyperactivated after LPS challenge. Both inhibitory and activating functions have been reported for CD96*.

Notably, a few studies have analyzed the expression of the main Tc-ICs, PD1, CTLA-4, TIM-3, LAG3, TIGIT, and VISTA in freshly isolated and *in vitro* activated/expanded NK cells in humans. From these studies it has been consistently reported that both TIM-3 and TIGIT are expressed by naïve as well as activated NK cells (69–72). Notably, TIM-3 and TIGIT has been found to regulate NK cell tolerance *in vivo* suggesting that they might work as a canonical IC in NK cells (15). Indeed, TIM3 regulates NK cell-tolerance during pregnancy (73) and TIGIT regulates liver regeneration (74). In contrast, it has not been reported yet if other Tc cell-ICs, like VISTA, PD1, CTLA4, B7-H3/H4, or LAG3, contribute to NK cell tolerance and would act as canonical ICs of NK cells (Figure 1).

VISTA has not been found in healthy human NK cells even after activation (75) and the expression of CTLA4 in human NK cells is not clear. It was found CTLA-4 in activated mouse NK cells (76) and later on another group reported that human NK cells expressed intracellular CTLA4 (77). However, the relevance of this finding is not clear since it was previously shown that human NK cells do not express CTLA4 and are not co-stimulated by the CD28/CTLA4 pathway (78). In agreement with the later study, we have not found CTLA4 in the membrane of cytokine-activated human NK cells (Lanuza et al., in preparation). Regarding other i ICs like B7-H3 and B7-H4, only B7-H3 has been found in activated healthy NK cells (79). However, although B7-H3 seems to be a negative regulator of both Tc and NK cell activity in mouse cancer models (80), in contrast to T cells, it does not seem to be involved in the regulation of NK cell tolerance (79). Finally, inhibitory LAG3 is expressed by activated NK cells (81) although it has not been found to contribute to the regulation of NK cell tolerance (15) and/or NK cell cytotoxic activity (82). A recent work found out that LAG3 was expressed at very low levels in activated NK cells, but its expression was substantially increased in the adaptive NK cell subset chronically exposed to NKG2C ligands like antibodies or CMV-infected cells (36). Importantly, these cells presented low activity against tumor cells, suggesting that memory-like NK cells from healthy donors previously infected with CMV, might become exhausted in case of CMV reactivation or after exposure to a new CMV infection in patients transferred with this NK cell subset. Notably, all experiments were performed in the presence of IL15 suggesting that chronically activation of adaptive NK cells in the presence of NKG2C ligands might require IL15 for induction of PD1 and LAG3 expression. However, the specific role of IL15 in this process is not completely clarified yet since the analyses of chronical activation with other cytokines like IL21 was not performed.

This issue is of special interest in light of the different protocols to expand allogenic NK cells that commonly rely on IL15 presence and the recent findings suggesting that continuous stimulation by IL15 exhausts NK cells (83). Since this study did not analyse the expression of PD1 and LAG3 on NK cells, it will be important to find out whether long term expansion of NK cells in the presence of IL15 could render NK cell populations whose anti-tumoral activity might be restricted by PD1 and/or LAG3.

### Different Function of the PD1/PD-L1 Inhibitory Axes in the Regulation of NK Cells in Cancer vs. Healthy Individuals

It seems that only TIM3 and TIGIT are clearly expressed and act as conventional ICs in NK cells (15, 69–72) and, thus, they might contribute to the regulation of NK cell anti-tumoral activity during adoptive allogeneic NK cell therapy. But, what about the golden boy of ICs in cancer immunotherapy, the PD1/PD-L1 inhibitory axes?

Recently, it has been shown that NK cells contribute to the efficacy of antibodies against the PD1/PD-L1 axes *in vivo* (84). This finding confirms that PD1 is not only inhibiting T cells in the cancer microenvironment but, in addition, it prevents NK cell anti-tumoral activity. Studies in humans have also suggested such a function since NK cells from cancer patients express increased levels of PD1, which correlates with a lower anti-tumoral activity (85–87).

However, and in contrast to NK cells isolated from cancer patients, the role of PD1/PD-L1 inhibitory axes in the anti-tumoral activity of NK cells from healthy donors is unclear. Indeed, its contribution to the anti-tumoral activity of allogeneic NK cells has not been clarified yet. In a recent study we have analyzed the role of PD /L1 inhibitory axes in the anti-tumoral activity of NK cells against colorectal cancer cells (88). Using colorectal cell lines expressing different levels of PDL1 expression we found that activated allogeneic NK cells can kill colorectal cancer cells irrespectively of PDL1 expression. This finding indicates that allogeneic healthy NK cells can overcome the PD1/PD-L1 inhibitory axes. However, since we did not analyse the effect of PD1/PD-L1 inhibitors, it is still possible that blocking of the PD1/PD-L1 axes increases the anti-tumoral activity of healthy allogeneic NK cells. If true, activated allogeneic NK cells should express PD1, an issue that still requires further clarification (89).

It should be noted here that in contrast to T cells, PD1 seems not to be involved in the regulation of NK cell tolerance, indicating that like in the case of CTLA4, LAG3, VISTA, or B7-H3/4, PD1 is not a conventional IC in NK cells. This is not surprising since the level of expression of PD1 in the membrane of naïve and activated NK cells from most healthy patients is generally very low in comparison with activated T cells or with NK cells from cancer patients (85–87, 90). In line with these findings, it has been recently found that a pool of intracellular PD1 is expressed at low level by both naïve and activated NK cells from healthy patients. Notably, the expression of this pool of cytosolic PD1 did not increase after *in vitro* cytokine activation and neither naïve nor activated NK cells expressed membrane PD1 (91). As indicated previously, a notable exception was recently reported by Pesce et al. who found that PD1 could be expressed at high levels in a subset of NK cells corresponding to fully mature cells, detectable in peripheral blood from around 25% healthy individuals who were serologically positive for cytomegalovirus (CMV) (16). This subset corresponded to CD56dim NK cells characterized by the absence of NKG2A and a high expression of CD57 and KIR, indicating an adaptive memory-like NK cell phenotype. This finding was confirmed later on by Merino et al., who reported a significant increase of PD1 (together with LAG3 as mentioned above) in the adaptive NK cell subset chronically activated by the NKG2C receptor, involved in CMV recognition (36).

Although it is not clear yet the significance of this finding, it merits special attention due to the special characteristics of adaptive memory-like NK cells. It has been found that in the context of viral infections adaptive NK cells specifically respond against the specific virus for which they were originally activated (5, 6). Thus, somehow it could be said that they are restricted by specific antigens/signals, although this restriction does seem to involve HLA-mediated antigen presentation (7). Thus, it could be speculated that similarly to T cells, the expression of PD1 and LAG3 in adaptive NK cells might be related to the regulation of antigen specificity, albeit this hypothesis will require experimental validation. In the context of tumor development, the role of antigen specificity in the generation and response of adaptive NK cells is not known.

Anyway, these findings suggest that like in the case of LAG3, PD1 expression could be upregulated in NK cells that have responded to CMV infection and thus, healthy NK cells from these individuals might be partially regulated by the PD1/PD-L1 inhibitory axes. A question that should be taken into account when designing protocols for adoptive NK cell therapy.

## Concluding Remarks and Future Perspectives

The emergence of ICIs like antibodies against the PD1/PD-L1 axes and CTLA4, or more recently against TIM3, LAG3, TIGIT, or PVRIG, has supposed a great advance in the treatment of very aggressive cancers like melanoma and lung carcinoma (92). These treatments mainly rely on the generation of robust cytotoxic T (Tc) cell responses against mutations/neoantigens present in cancer cells. Indeed, cancer has learnt to use the main T cell-related ICs, naturally evolved to control T cell activity and avoid autoimmunity, to overcome T cell-mediated recognition and destruction. However, several tumor types present low immunogenicity and do not respond to these therapies, remaining incurable. Even immunogenic cancers that initially respond to ICIs, frequently relapse due to the selection of poorly immunogenic cell clones and/or the apparition of alternative resistance mechanisms (92, 93). The combination of different ICIs like PD1/PD-L1 and CTLA4 inhibitors to overcome these limitations often increases toxicity and treatment has to be discontinued (92).

As an alternative, the use of NK cells isolated from haploidentical healthy patients and activated *in vitro*, allogeneic NK cells, presents several advantages that might help to overcome the limitations of ICI immunotherapy and to treat patients that do not respond to ICIs. First, alloreactivity due to KIR-HLA-I mismatch overcome the main NK cell-ICs, KIRs, and thus the inhibitory signals by HLA-I expressing tumor cells (52–54). Second, NK cell tolerance and NK cell-mediated cancer and infection immunosurveillance are mainly regulated by ICs different from T cell-related ICs (3, 4, 8) (Figure 1). Thus, NK cells developed in a healthy environment and activated *in vitro* should not be affected by cancer immune evasion based on ICs of T cells. Canonical inhibitory NK cell-ICs include the KIR family, NKG2A and ILT2 and among other ICs, only TIM3 and TIGIT have been clearly found to be involved in NK cell function in healthy individuals (3, 4, 8). Thus, the most potent ICs found in cancer, CTLA4, LAG3, and the PD1/PD-L1 axes should not affect the cytotoxic anti-tumoral activity of allogeneic NK cells. Finally, in contrast to Tc cells, which strictly depends on the activating signals transduced by the TCR after recognizing antigens presented by HLA-I, NK cells present a vast array of receptors that transduce activating signals like activating KIRs, NCRs of NKG2D/C. Thus, reduction of the inhibitory signals in the tumor microenvironment employing healthy allogeneic NK cells that express few T cell-ICs should unmask cancer cells to be efficiently eliminated by transferred activated NK cells.

However, in order to develop efficient protocols based on allogeneic NK cells to treat solid and hematological tumors and generate activated NK cells resistant to ICs, some limitations and open questions needs to be solved. Among them, we propose some key questions that, from our point of view, are important and deserve further experimental validation.

1. It will be important to find out if healthy *in vitro* activated allogeneic NK cells are induced to express other T cell-ICs like PD1, CTLA4, VISTA or LAG3 by cytokines present in the tumor microenvironment. On this regard it was recently found that PD1 was induced by TGF β in T cells (94, 95). It will be interesting to analyse if a similar effect is found in healthy activated NK cells.
2. It will be interesting to find out if specific cytokines or other stimulus are able to regulate IC expression on NK cells activated/expanded *in vitro*. In this regard IL15 was recently found to exhaust NK cells in long term *in vitro* cultures, although the expression of ICs like PD1 or LAG3 was not analyzed (83). Thus, in order to predict its efficacy on a personalized way, it should be required to analyse the profile of T cell-IC expression on *in vitro* expanded NK cells used for adoptive cell transfer. Depending on the results specific protocols for NK cell activation and expansion might be developed to minimize the expression of ICs on NK cells without affecting the expansion rate and cytotoxic potential.
3. Since it has been found increased PD1 and LAG3 expression in adaptive NK cells from CMV positive healthy individuals, it would be interesting to analyse if other T cell-ICs are also upregulated in these individuals. This finding also suggests that selection of CMV negative NK cell donors might help to generate PD1/LAG3 insensitive NK cells.
4. Combination of antibodies against tumor antigens or against soluble ligands that inhibits activating NK cell receptors with allogeneic NK cells should augment the activating signals on allogeneic NK cells and help to treat cancers that express high levels of ligands for emerging inhibitory NK-ICs like TIM3 or TIGIT.
5. As indicated, it has been found that low levels of CTLA4 and PD1 are expressed in activated human healthy NK cell intracellularly. Thus, it will be important to find out the stimulus that might mobilize PD1 and/or CTLA4 to the cell membrane and if this low level of expression is enough to inhibit NK cell-mediated elimination of cancer cells in the presence of the respective inhibitory ligands. This will be important to predict if intracellular PD1 will be expressed on the cell membrane of NK cells once they reach the tumor microenvironment.
6. It will be important to clarify if the contribution of host NK cells to immunotherapy with ICIs is due to a direct effect of ICI to NK cell activation or to a synergic effect between NK cells and Tc cells released from the IC breaks. On this way more effective protocols combining allogeneic NK cells and ICIs could be developed.
7. It will be important to find out the role of specific NK cell-ICs in tolerance regulation. On this way genetic manipulation of ICs in NK cells or combination of allogeneic NK cells with ICIs could be rationally developed to increase the efficacy of adoptive allogeneic NK cell transfer in a safe way.

Pending of validate experimentally all these hypotheses and in light of the available experimental evidences, it could be concluded that the regulation of the anti-tumoral activity of most subsets of allogeneic activated NK cells by T cell-ICs like CTLA4, PD1, VISTA, LAG3, or B7H3/4 is low. This might be an advantage to use adoptive allogeneic NK cell therapy to treat cancer types that despite presenting high immunogenicity and/or high levels of ICs do not respond to ICIs. Thus, efforts should be focused in solving other questions that might affect the efficacy of allogeneic NK cell therapy like migration and infiltration into solid tumors or the presence of other immunosuppressive factors in the tumor microenvironment like immunosuppressive cytokines or metabolic negative regulators.

## Author Contributions

All authors have contributed to the writing and editing of the manuscript. JP, AR-L, and PL designed the review. JP designed the figures.

### Conflict of Interest

JP participates in research grants from Bristol-Myers Squibb and Gilead Sciences and has received speaker honorary from Gilead Sciences, Pfizer and Fundación Biomédica Miguel Servet. The remaining authors declare that the research was conducted in the absence of any commercial or financial relationships that could be construed as a potential conflict of interest.

## References

[1] Cooper MA Fehniger TA Caligiuri MA. The biology of human natural killer-cell subsets. Trends Immunol. (2001) 22:633–40. 10.1016/S1471-4906(01)02060-9 11698225

[2] Michel T Poli A Cuapio A Briquemont B Iserentant G Ollert M. Human CD56bright NK Cells: An update. J Immunol. (2016) 196:2923–31. 10.4049/jimmunol.1502570 26994304

[3] Watzl C Chapter Five: How to trigger a killer: modulation of natural killer cell reactivity on many levels. In: Alt FW editor. Advances in Immunology. Cambridge: Academic Press (2014). p. 137–70. 10.1016/B978-0-12-800147-9.00005-4 25175775

[4] Long EO Kim HS Liu D Peterson ME Rajagopalan S. Controlling natural killer cell responses: integration of signals for activation and inhibition. Annu Rev Immunol. (2013) 31:227–58. 10.1146/annurev-immunol-020711-075005 23516982 PMC3868343

[5] O'Sullivan TE Sun JC Lanier LL Natural killer cell memory. Immunity. (2015) 43:634–45. 10.1016/j.immuni.2015.09.013 26488815 PMC4621966

[6] Min-Oo G Kamimura Y Hendricks DW Nabekura T Lanier LL. Natural killer cells: walking three paths down memory lane. Trends Immunol. (2013) 34:251–8. 10.1016/j.it.2013.02.005 23499559 PMC3674190

[7] Jamieson AM Isnard P Dorfman JR Coles MC Raulet DH. Turnover and proliferation of NK cells in steady state and lymphopenic conditions. J Immunol. (2004) 172:864. 10.4049/jimmunol.172.2.864 14707057

[8] Held W Kijima M Angelov G Bessoles SP. The function of natural killer cells: education, reminders and some good memories. Curr Opin Immunol. (2011) 23:228–33. 10.1016/j.coi.2010.11.008 21159498

[9] Sivori S Falco M Marcenaro E Parolini S Biassoni R Bottino C. Early expression of triggering receptors and regulatory role of 2B4 in human natural killer cell precursors undergoing in vitro differentiation. Proc Natl Acad Sci USA. (2002) 99:4526–31. 10.1073/pnas.072065999 11917118 PMC123681

[10] Shifrin N Raulet DH Ardolino M. NK cell self tolerance, responsiveness and missing self recognition. Semin Immunol. (2014) 26:138–44. 10.1016/j.smim.2014.02.007 24629893 PMC3984600

[11] Assarsson E Kambayashi T Persson CM Chambers BJ Ljunggren HG. 2B4/CD48-mediated regulation of lymphocyte activation and function. J Immunol. (2005) 175:2045. 10.4049/jimmunol.175.4.2045 16081768

[12] Mooney JM Klem J Wülfing C Mijares LA Schwartzberg PL Bennett M. The murine NK receptor 2B4 (CD244) exhibits inhibitory function independent of signaling lymphocytic activation molecule-associated protein expression. J Immunol. (2004) 173:3953. 10.4049/jimmunol.173.6.3953 15356144

[13] Parolini S Bottino C Falco M Augugliaro R Giliani S Franceschini R. X-linked lymphoproliferative disease. 2B4 molecules displaying inhibitory rather than activating function are responsible for the inability of natural killer cells to kill Epstein-Barr virus-infected cells. J Exp Med. (2000) 192:337–46. 10.1084/jem.192.3.337 10934222 PMC2193227

[14] Sivori S Vacca P Del Zotto G Munari E Mingari MC Moretta L. Human NK cells: surface receptors, inhibitory checkpoints, and translational applications. Cell Mol Immunol. (2019) 16:430–41. 10.1038/s41423-019-0206-4 30778167 PMC6474200

[15] Anderson AC Joller N Kuchroo VK. Lag-3, Tim-3, and TIGIT: co-inhibitory receptors with specialized functions in immune regulation. Immunity. (2016) 44:989–1004. 10.1016/j.immuni.2016.05.001 27192565 PMC4942846

[16] Pesce S Greppi M Tabellini G Rampinelli F Parolini S Olive D. Identification of a subset of human natural killer cells expressing high levels of programmed death 1: a phenotypic and functional characterization. J Allergy Clin Immunol. (2017) 139:335–46.e3. 10.1016/j.jaci.2016.04.025 27372564

[17] Della Chiesa M Pesce S Muccio L Carlomagno S Sivori S Moretta A. Features of memory-like and PD-1(+) human NK cell subsets. Front Immunol. (2016) 7:351. 10.3389/fimmu.2016.00351 27683578 PMC5021715

[18] Pérez-Villar JJ Melero I Navarro F Carretero M Bellón T Llano M. The CD94/NKG2-A inhibitory receptor complex is involved in natural killer cell-mediated recognition of cells expressing HLA-G1. J Immunol. (1997) 158:5736. 9190923

[19] Braud VM Allan DSJ O'Callaghan CA Söderström K D'Andrea A Ogg GS. HLA-E binds to natural killer cell receptors CD94/NKG2A, B and C. Nature. (1998) 391:795–9. 10.1038/35869 9486650

[20] Carretero M Cantoni C Bellón T Bottino C Biassoni R Rodríguez A. The CD94 and NKG2-A C-type lectins covalently assemble to form a natural killer cell inhibitory receptor for HLA class I molecules. Eur J Immunol. (1997) 27:563–7. 10.1002/eji.1830270230 9045931

[21] Kruse PH Matta J Ugolini S Vivier E. Natural cytotoxicity receptors and their ligands. Immunol Cell Biol. (2014) 92:221–9. 10.1038/icb.2013.98 24366519

[22] Bauer S Groh V Wu J Steinle A Phillips JH Lanier LL. Activation of NK cells and T cells by NKG2D, a receptor for stress-inducible MICA. Science. (1999) 285:727–29. 10.1126/science.285.5428.727 10426993

[23] Thielens A Vivier E Romagné F. NK cell MHC class I specific receptors (KIR): from biology to clinical intervention. Curr Opin Immunol. (2012) 24:239–45. 10.1016/j.coi.2012.01.001 22264929

[24] Bartel Y Bauer B Steinle A. Modulation of NK cell function by genetically coupled C-type lectin-like receptor/ligand pairs encoded in the human natural killer gene complex. Front Immunol. (2013) 4:362. 10.3389/fimmu.2013.00362 24223577 PMC3819593

[25] Moretta A Bottino C Vitale M Pende D Cantoni C Mingari MC. Activating receptors and coreceptors involved in human natural killer cell-mediated cytolysis. Ann Rev Immunol. (2001) 19:197–223. 10.1146/annurev.immunol.19.1.197 11244035

[26] Vitale M Bottino C Sivori S Sanseverino L Castriconi R Marcenaro E. NKp44, a novel triggering surface molecule specifically expressed by activated natural killer cells, is involved in non-major histocompatibility complex-restricted tumor cell lysis. J Exp Med. (1998) 187:2065–72. 10.1084/jem.187.12.2065 9625766 PMC2212362

[27] Parodi M Favoreel H Candiano G Gaggero S Sivori S Mingari MC. NKp44-NKp44 ligand interactions in the regulation of natural killer cells and other innate lymphoid cells in humans. Front Immunol. (2019) 10:719. 10.3389/fimmu.2019.00719 31024551 PMC6465645

[28] Guillerey C Huntington ND Smyth MJ. Targeting natural killer cells in cancer immunotherapy. Nat Immunol. (2016) 17:1025–36. 10.1038/ni.3518 27540992

[29] Rosental B Brusilovsky M Hadad U Oz D Appel MY Afergan F. Proliferating cell nuclear antigen is a novel inhibitory ligand for the natural cytotoxicity receptor NKp44. J Immunol. (2011) 187:5693–702. 10.4049/jimmunol.1102267 22021614 PMC3269963

[30] Kundu K Ghosh S Sarkar R Edri A Brusilovsky M Gershoni-Yahalom O. Inhibition of the NKp44-PCNA immune checkpoint using a mAb to PCNA. Cancer Immunol Res. (2019) 7:1120. 10.1158/2326-6066.CIR-19-0023 31164357 PMC7233522

[31] Correia MP Stojanovic A Bauer K Juraeva D Tykocinski L-O Lorenz H-M. Distinct human circulating NKp30(+)FcεRIγ(+)CD8(+) T cell population exhibiting high natural killer-like antitumor potential. Proc Natl Acad Sci USA. (2018) 115:E5980–9. 10.1073/pnas.1720564115 29895693 PMC6042091

[32] Wensveen FM Jelenčić V Polić B. NKG2D: a master regulator of immune cell responsiveness. Front Immunol. (2018) 9:441. 10.3389/fimmu.2018.00441 29568297 PMC5852076

[33] Braud VM Aldemir H Breart B Ferlin WG. Expression of CD94–NKG2A inhibitory receptor is restricted to a subset of CD8+ T cells. Trends Immunol. (2003) 24:162–4. 10.1016/S1471-4906(03)00064-4 12697440

[34] van Montfoort N Borst L Korrer MJ Sluijter M Marijt KA Santegoets SJ. NKG2A Blockade potentiates CD8 T cell immunity induced by cancer vaccines. Cell. (2018) 175:1744–55.e15. 10.1016/j.cell.2018.10.028 30503208 PMC6354585

[35] André P Denis C Soulas C Bourbon-Caillet C Lopez J Arnoux T. Anti-NKG2A mAb Is a checkpoint inhibitor that promotes anti-tumor immunity by unleashing both T and NK cells. Cell. (2018) 175:1731–43.e13. 10.1016/j.cell.2018.10.014 30503213 PMC6292840

[36] Merino A Zhang B Dougherty P Luo X Wang J Blazar BR Chronic stimulation drives human NK cell dysfunction and epigenetic reprograming. J Clin Investig. (2019) 129:3770–85. 10.1172/JCI125916 PMC6715389 31211698

[37] Liang S Zhang W Horuzsko A. Human ILT2 receptor associates with murine MHC class I molecules in vivo and impairs T cell function. Eur J Immunol. (2006) 36:2457–71. 10.1002/eji.200636031 16897816

[38] Sanchez-Correa B Valhondo I Hassouneh F Lopez-Sejas N Pera A Bergua JM. DNAM-1 and the TIGIT/PVRIG/TACTILE Axis: novel immune checkpoints for natural killer cell-based cancer immunotherapy. Cancers. (2019) 11:877. 10.3390/cancers11060877 31234588 PMC6628015

[39] Muntasell A Ochoa MC Cordeiro L Berraondo P López-Díaz de Cerio A Cabo M. Targeting NK-cell checkpoints for cancer immunotherapy. Curr Opin Immunol. (2017) 45:73–81. 10.1016/j.coi.2017.01.003 28236750

[40] Bryceson YT March ME Ljunggren H-G Long EO. Activation, coactivation, and costimulation of resting human natural killer cells. Immunol Rev. (2006) 214:73–91. 10.1111/j.1600-065X.2006.00457.x 17100877 PMC3845883

[41] Kohrt HE Thielens A Marabelle A Sagiv-Barfi I Sola C Chanuc F. Anti-KIR antibody enhancement of anti-lymphoma activity of natural killer cells as monotherapy and in combination with anti-CD20 antibodies. Blood. (2014) 123:678–86. 10.1182/blood-2013-08-519199 24326534 PMC3907754

[42] Sola C Chanuc F Thielens A Fuseri N Morel Y Bléry M Anti-tumoral efficacy of therapeutic human anti-KIR antibody (Lirilumab/BMS-986015/IPH2102) in a preclinical xenograft tumor model. J Immunother Cancer. (2013) 1(Suppl 1):P40 10.1186/2051-1426-1-S1-P40

[43] Yalniz FF Daver N Rezvani K Kornblau S Ohanian M Borthakur G A Pilot trial of lirilumab with or without azacitidine for patients with Myelodysplastic Syndrome. Clin Lymphoma Myeloma Leuk. (2018) 18:658–63.e2. 10.1016/j.clml.2018.06.011 30001986 PMC6750214

[44] He Y Liu S Mattei J Bunn PA Jr Zhou C Chan D. The combination of anti-KIR monoclonal antibodies with anti-PD-1/PD-L1 monoclonal antibodies could be a critical breakthrough in overcoming tumor immune escape in NSCLC. Drug Design Dev Ther. (2018) 12:981–6. 10.2147/DDDT.S163304 29731605 PMC5923225

[45] Gauthier L Morel A Anceriz N Rossi B Blanchard-Alvarez A Grondin G. Multifunctional Natural Killer cell engagers targeting NKp46 trigger protective tumor immunity. Cell. (2019) 177:1701–13.e16. 10.1016/j.cell.2019.04.041 31155232

[46] Reiners KS Kessler J Sauer M Rothe A Hansen HP Reusch U. Rescue of impaired NK cell activity in hodgkin lymphoma with bispecific antibodies *in vitro* and in patients. Mol Ther. (2013) 21:895–903. 10.1038/mt.2013.14 23459515 PMC3616527

[47] Carlsten M Korde N Kotecha R Reger R Bor S Kazandjian D. Checkpoint inhibition of KIR2D with the monoclonal antibody IPH2101 induces contraction and hyporesponsiveness of NK cells in patients with myeloma. Clin Cancer Res. (2016) 22:5211. 10.1158/1078-0432.CCR-16-1108 27307594 PMC8638787

[48] Felices M Miller JS. Targeting KIR blockade in multiple myeloma: trouble in checkpoint paradise? Clin Cancer Res. (2016) 22:5161–3. 10.1158/1078-0432.CCR-16-1582 27430580 PMC5093060

[49] Gras Navarro A Björklund AT Chekenya M. Therapeutic potential and challenges of natural killer cells in treatment of solid tumors. Front Immunol. (2015) 6:202. 10.3389/fimmu.2015.00202 25972872 PMC4413815

[50] Kärre K. NK cells, MHC class I molecules and the missing self. Scand J Immunol. (2002) 55:221–8. 10.1046/j.1365-3083.2002.01053.x 11940227

[51] Farag SS Fehniger TA Ruggeri L Velardi A Caligiuri MA. Natural killer cell receptors: new biology and insights into the graft-versus-leukemia effect. Blood. (2002) 100:1935–47. 10.1182/blood-2002-02-0350 12200350

[52] Ruggeri L Capanni M Casucci M Volpi I Tosti A Perruccio K. Role of natural killer cell alloreactivity in HLA-mismatched hematopoietic stem cell transplantation. Blood. (1999) 94:333–9. 10.1182/blood.V94.1.333.413a31_333_339 10381530

[53] Ruggeri L Capanni M Urbani E Perruccio K Shlomchik WD Tosti A. Effectiveness of donor Natural Killer cell alloreactivity in mismatched hematopoietic transplants. Science. (2002) 295:2097–100. 10.1126/science.1068440 11896281

[54] Miller JS Cooley S Parham P Farag SS Verneris MR McQueen KL. Missing KIR ligands are associated with less relapse and increased graft-versus-host disease (GVHD) following unrelated donor allogeneic HCT. Blood. (2007) 109:5058–61. 10.1182/blood-2007-01-065383 17317850 PMC1885526

[55] Davis ZB Felices M Verneris MR Miller JS. Natural Killer cell adoptive transfer therapy: exploiting the first line of defense against cancer. Cancer J. (2015) 21:486–91. 10.1097/PPO.0000000000000156 26588681 PMC4763946

[56] Moretta L Pietra G Vacca P Pende D Moretta F Bertaina A. Human NK cells: from surface receptors to clinical applications. Immunol Lett. (2016) 178:15–9. 10.1016/j.imlet.2016.05.007 27185471

[57] Lim O Jung MY Hwang YK Shin E-C. Present and future of allogeneic natural killer cell therapy. Front Immunol. (2015) 6:286. 10.3389/fimmu.2015.00286 26089823 PMC4453480

[58] Cichocki F Verneris MR Cooley S Bachanova V Brunstein CG Blazar BR. The past, present, and future of NK cells in hematopoietic cell transplantation and adoptive transfer. Curr Top Microbiol Immunol. (2016) 395:225–43. 10.1007/82_2015_445 26037048 PMC5870762

[59] Becker PSA Suck G Nowakowska P Ullrich E Seifried E Bader P. Selection and expansion of natural killer cells for NK cell-based immunotherapy. Cancer Immunol Immunother. (2016) 65:477–84. 10.1007/s00262-016-1792-y 26810567 PMC4826432

[60] Zhang Q Vignali DAA. Co-stimulatory and co-inhibitory pathways in autoimmunity. Immunity. (2016) 44:1034–51. 10.1016/j.immuni.2016.04.017 27192568 PMC4873959

[61] Greenwald RJ Freeman GJ Sharpe AH. The B7 family revisited. Ann Rev Immunol. (2004) 23:515–48. 10.1146/annurev.immunol.23.021704.115611 15771580

[62] Waterhouse P Penninger JM Timms E Wakeham A Shahinian A Lee KP. Lymphoproliferative disorders with early lethality in mice deficient in Ctla-4. Science. (1995) 270:985–8. 10.1126/science.270.5238.985 7481803

[63] Tivol EA Borriello FA Schweitzer N Lynch WP Bluestone JA Sharpe AH. Loss of CTLA-4 leads to massive lymphoproliferation and fatal multiorgan tissue destruction, revealing a critical negative regulatory role of CTLA-4. Immunity. (1995) 3:541–7. 10.1016/1074-7613(95)90125-6 7584144

[64] Nishimura H Nose M Hiai H Minato N Honjo T. Development of lupus-like autoimmune diseases by disruption of the PD-1 gene encoding an ITIM motif-carrying immunoreceptor. Immunity. (1999) 11:141–51. 10.1016/S1074-7613(00)80089-8 10485649

[65] Krummel MF Allison JP. CD28 and CTLA-4 have opposing effects on the response of T cells to stimulation. J Exp Med. (1995) 182:459–65. 10.1084/jem.182.2.459 7543139 PMC2192127

[66] Okazaki T Honjo T. PD-1 and PD-1 ligands: from discovery to clinical application. Int Immunol. (2007) 19:813–24. 10.1093/intimm/dxm057 17606980

[67] Pardoll DM. The blockade of immune checkpoints in cancer immunotherapy. Nat Rev Cancer. (2012) 12:252–64. 10.1038/nrc3239 22437870 PMC4856023

[68] Souza-Fonseca-Guimaraes F Cursons J Huntington ND. The emergence of natural killer cells as a major target in cancer immunotherapy. Trends Immunol. (2019) 40:142–58. 10.1016/j.it.2018.12.003 30639050

[69] Ndhlovu LC Lopez-Vergès S Barbour JD Jones RB Jha AR Long BR. Tim-3 marks human natural killer cell maturation and suppresses cell-mediated cytotoxicity. Blood. (2012) 119:3734–43. 10.1182/blood-2011-11-392951 22383801 PMC3335380

[70] Gleason MK Lenvik TR McCullar V Felices M O'Brien MS Cooley SA. Tim-3 is an inducible human natural killer cell receptor that enhances interferon gamma production in response to galectin-9. Blood. (2012) 119:3064–72. 10.1182/blood-2011-06-360321 22323453 PMC3321868

[71] Stanietsky N Simic H Arapovic J Toporik A Levy O Novik A. The interaction of TIGIT with PVR and PVRL2 inhibits human NK cell cytotoxicity. Proc Natl Acad Sci USA. (2009) 106:17858–63. 10.1073/pnas.0903474106 19815499 PMC2764881

[72] Wang F Hou H Wu S Tang Q Liu W Huang M. TIGIT expression levels on human NK cells correlate with functional heterogeneity among healthy individuals. Eur J Immunol. (2015) 45:2886–97. 10.1002/eji.201545480 26171588

[73] Li Y Zhang J Zhang D Hong X Tao Y Wang S. Tim-3 signaling in peripheral NK cells promotes maternal-fetal immune tolerance and alleviates pregnancy loss. Science Signaling. (2017) 10:eaah4323. 10.1126/scisignal.aah4323 28951537

[74] Bi J Zheng X Chen Y Wei H Sun R Tian Z. TIGIT safeguards liver regeneration through regulating natural killer cell-hepatocyte crosstalk. Hepatology. (2014) 60:1389–98. 10.1002/hep.27245 24912841

[75] Lines JL Pantazi E Mak J Sempere LF Wang L O'Connell S. VISTA is an immune checkpoint molecule for human T cells. Cancer Res. (2014) 74:1924–32. 10.1158/0008-5472.CAN-13-1504 24691993 PMC3979527

[76] Stojanovic A Fiegler N Brunner-Weinzierl M Cerwenka A. CTLA-4 is expressed by activated mouse NK cells and inhibits NK Cell IFN-γ production in response to mature dendritic cells. J Immunol. (2014) 192:4184–91. 10.4049/jimmunol.1302091 24688023

[77] Lougaris V Tabellini G Baronio M Patrizi O Gazzurelli L Mitsuiki N. CTLA-4 regulates human Natural Killer cell effector functions. Clin Immunol. (2018) 194:43–5. 10.1016/j.clim.2018.06.010 29966715

[78] Lang S Vujanovic NL Wollenberg B Whiteside TL. Absence of B7.1-CD28/CTLA-4-mediated co-stimulation in human NK cells. Eur J Immunol. (1998) 28:780–6. 9541571 10.1002/(SICI)1521-4141(199803)28:03<780::AID-IMMU780>3.0.CO;2-8

[79] Yi KH Chen L. Fine tuning the immune response through B7-H3 and B7-H4. Immunol Rev. (2009) 229:145–51. 10.1111/j.1600-065X.2009.00768.x 19426220 PMC2696224

[80] Lee J-H Tammela T Hofree M Choi J Marjanovic ND Han S. Anatomically and functionally distinct lung mesenchymal populations marked by Lgr5 and Lgr6. Cell. (2017) 170:1149–63.e12. 10.1016/j.cell.2017.07.028 28886383 PMC5607351

[81] Triebel F Jitsukawa S Baixeras E Roman-Roman S Genevee C Viegas-Pequignot E. LAG-3, a novel lymphocyte activation gene closely related to CD4. J Exp Med. (1990) 171:1393–405. 10.1084/jem.171.5.1393 1692078 PMC2187904

[82] Huard B Tournier M Triebel F LAG-3 does not define a specific mode of natural killing in human. Immunol Lett. (1998) 61:109–12. 10.1016/S0165-2478(97)00170-3 9657262

[83] Felices M Lenvik AJ McElmurry R Chu S Hinderlie P Bendzick L. Continuous treatment with IL-15 exhausts human NK cells via a metabolic defect. JCI Insight. (2018) 3:e96219. 10.1172/jci.insight.96219 29415897 PMC5821201

[84] Hsu J Hodgins JJ Marathe M Nicolai CJ Bourgeois-Daigneault M-C Trevino TN. Contribution of NK cells to immunotherapy mediated by PD-1/PD-L1 blockade. J Clin Investig. (2018) 128:4654–68. 10.1172/JCI99317 30198904 PMC6159991

[85] Liu Y Cheng Y Xu Y Wang Z Du X Li C. Increased expression of programmed cell death protein 1 on NK cells inhibits NK-cell-mediated anti-tumor function and indicates poor prognosis in digestive cancers. Oncogene. (2017) 36:6143–53. 10.1038/onc.2017.209 28692048 PMC5671935

[86] Benson DM Jr Bakan CE Mishra A Hofmeister CC Efebera Y Becknell B. The PD-1/PD-L1 axis modulates the natural killer cell versus multiple myeloma effect: a therapeutic target for CT-011, a novel monoclonal anti-PD-1 antibody. Blood. (2010) 116:2286–94. 10.1182/blood-2010-02-271874 20460501 PMC3490105

[87] Concha-Benavente F Kansy B Moskovitz J Moy J Chandran U Ferris RL. PD-L1 mediates dysfunction in activated PD-1+ NK cells in head and neck cancer patients. Cancer Immunol Res. (2018) 6:1548–60. 10.1158/2326-6066.CIR-18-0062 30282672 PMC6512340

[88] Lanuza PM Vigueras A Olivan S Prats AC Costas S Llamazares G. Activated human primary NK cells efficiently kill colorectal cancer cells in 3D spheroid cultures irrespectively of the level of PD-L1 expression. Oncoimmunology. (2017) 7:e1395123. 10.1080/2162402X.2017.1395123 29632716 PMC5889279

[89] Pesce S Greppi M Grossi F Del Zotto G Moretta L Sivori S. PD/1-PD-Ls Checkpoint: insight on the potential role of NK cells. Front Immunol. (2019) 10:1242. 10.3389/fimmu.2019.01242 31214193 PMC6557993

[90] Beldi-Ferchiou A Lambert M Dogniaux S Vély F Vivier E Olive D. PD-1 mediates functional exhaustion of activated NK cells in patients with Kaposi sarcoma. Oncotarget. (2016) 7:72961–77. 10.18632/oncotarget.12150 27662664 PMC5341956

[91] Mariotti FR Petrini S Ingegnere T Tumino N Besi F Scordamaglia F. PD-1 in human NK cells: evidence of cytoplasmic mRNA and protein expression. OncoImmunology. (2018) 8:1557030. 10.1080/2162402X.2018.1557030 30723590 PMC6350684

[92] Topalian SL Drake CG Pardoll DM. Immune checkpoint blockade: a common denominator approach to cancer therapy. Cancer Cell. (2015) 27:450–61. 10.1016/j.ccell.2015.03.001 25858804 PMC4400238

[93] Park Y-J Kuen D-S Chung Y. Future prospects of immune checkpoint blockade in cancer: from response prediction to overcoming resistance. Exp Mol Med. (2018) 50:109. 10.1038/s12276-018-0130-1 30135516 PMC6105674

[94] Baas M Besançon A Goncalves T Valette F Yagita H Sawitzki B. TGFβ-dependent expression of PD-1 and PD-L1 controls CD8+ T cell anergy in transplant tolerance. eLife. (2016) 5:e08133. 10.7554/eLife.08133 26824266 PMC4749558

[95] Park BV Freeman ZT Ghasemzadeh A Chattergoon MA Rutebemberwa A Steigner J TGF-β1-mediated Smad3 enhances PD-1 expression on antigen-specific T cells in cancer. Cancer Discov. (2016) 6:1366–81. 10.1158/2159-8290.CD-15-1347 27683557 PMC5295786

